# African Swine Fever Virus DP71L Protein Inhibits Dextran Sulfate Sodium (DSS)-Induced Murine Colitis

**DOI:** 10.3390/v18091016

**Published:** 2026-09-14

**Authors:** Xiaofeng Nian, Zhiyu Li, Yonghua Ma, Zifan Wang, Zilin Qiao, Zhaxi Yingpai, Weiwei Chai, Xiaofang Luo, Penghui Guo

**Affiliations:** 1 School of Life Science and Engineering, Northwest Minzu University, Lanzhou 730030, China; 2 China-Malaysia National Joint Laboratory, Biomedical Research Center, Engineering Research Center of Key Technology and Industrialization of Cell-Based Vaccine, Ministry of Education, Gansu Tech Innovation Center of Animal Cell, Key Laboratory of Biotechnology and Bioengineering of State Ethnic Affairs Commission, Northwest Minzu University, Lanzhou 730030, China; 185212768@xbmu.edu.cn (Z.W.);; 3 College of Veterinary Medicine, Gansu Agriculture University, Lanzhou 730070, China; 4 School of Bioengineering, Northwest Minzu University, Lanzhou 730030, China; 5 Experimental Teaching Department, Northwest Minzu University, Lanzhou 730030, China

**Keywords:** inflammatory bowel disease, dextran sulfate sodium, African swine fever virus, DP71L, immunosuppressant

## Abstract

The functions of most proteins encoded by the African swine fever virus (ASFV) remain largely unknown, although several have been reported to possess immunomodulatory properties. Among these, we identified that the DP71L protein exerts an inhibitory effect on inflammatory bowel disease. To investigate its protective role in murine colitis, we constructed and expressed a recombinant DP71L protein. Colitis was induced in mice using DSS, and the effects of DP71L treatment were evaluated by assessing histopathological changes, inflammatory cytokine profiles, oxidative stress markers, colonic tissue pathology, and the expression of tight-junction proteins (claudin-1, occludin, and ZO-1). Our results showed that DP71L intervention significantly attenuated body weight loss and organ damage and ameliorated DSS-induced colonic histopathological injury. Moreover, DP71L treatment markedly increased superoxide dismutase (SOD) activity and reduced malondialdehyde (MDA) content in colonic tissues. Mechanistically, DP71L suppressed both DSS-induced NF-κB and JAK-STAT activation and concurrently inhibited DSS-induced epithelial cell apoptosis. These events likely underlie the observed reduction in pro-inflammatory cytokines (IL-1β, IL-6, IFN-γ, and TNF-α) and the restoration of tight-junction protein expression, as DP71L treatment effectively prevented DSS-induced downregulation of claudin-1, occludin, and ZO-1, while also promoting the anti-inflammatory cytokines IL-10 and TGF-β. Collectively, our findings demonstrate that DP71L effectively inhibits the progression of murine colitis through coordinated anti-inflammatory and anti-apoptotic mechanisms. This study suggests that DP71L may function as a potential immunosuppressant, opening new avenues for the application of viral proteins in the treatment of immune-related disorders.

## 1. Introduction

Inflammatory bowel disease (IBD) is a chronic, relapsing immune-mediated disorder of the gastrointestinal tract, encompassing two major forms: Crohn’s disease (CD) and ulcerative colitis (UC). UC is characterized by continuous, mucosa-restricted inflammation that originates in the rectum and extends proximally, involving diffuse mucosal damage, crypt abscesses, goblet cell depletion, and infiltration by lymphocytes, plasma cells, and eosinophils [1]. Over the past century, the global burden of IBD has escalated substantially: from 1920 to 2010, the average annual incidence of CD rose from 1.2% to 23.3%, and that of UC from 2.4% to 18.1%, with rapidly increasing rates now observed in South America, Eastern Europe, Asia, and Africa [2]. Currently, IBD affects an estimated 6 million people worldwide, and its prevalence is also rising in developing nations such as China. Despite this growing public health challenge, conventional therapies—including 5-aminosalicylates, surgery, dietary interventions, probiotics, and fecal microbiota transplantation—often yield suboptimal outcomes, underscoring an urgent need for more effective and targeted treatments [3].

In response to this therapeutic gap, immunomodulatory agents that precisely interfere with key inflammatory signaling cascades, such as interleukin-23 (IL-23) and Janus kinase (JAK) inhibitors, are increasingly being evaluated in clinical trials for IBD [4]. The JAK-STAT (signal transducers and activators of transcription) pathway, in particular, has emerged as a critical node in IBD pathogenesis due to its role in transducing signals from multiple pro-inflammatory cytokines [5].

ASFV is a large cytoplasmic double-stranded DNA virus that causes near-100% fatal hemorrhagic fever in pigs; its genome encodes several immune-evasion proteins, including DP71L, which recruits protein phosphatase 1 (PP1) to dephosphorylate translation initiation factor eIF2α and inhibit the downstream pro-apoptotic factor C/EBP homologous protein (CHOP), thereby blocking host apoptosis—a mechanism functionally analogous to that of growth arrest and DNA damage-inducible protein 34 (GADD34) and HSV ICP34.5 [5,6,7,8,9,10,11]. Intriguingly, our observations indicate that the African swine fever virus (ASFV) nonstructural protein DP71L can suppress JAK-STAT signaling.

Given that dysregulated apoptosis and persistent JAK-STAT activation are intimately linked to inflammatory tissue damage in IBD, we hypothesized that DP71L might exert a therapeutic immunomodulatory effect beyond its canonical viral immune-evasion role. However, the potential application of this viral protein in autoimmune or chronic inflammatory conditions has not been previously explored. To test this hypothesis, we generated a recombinant DP71L protein and evaluated its efficacy in a DSS-induced murine colitis model. Our results demonstrate that DP71L administration significantly ameliorates colonic histopathological damage, improves oxidative stress parameters (elevated superoxide dismutase activity and reduced malondialdehyde content), and restores the expression of intestinal tight-junction proteins (claudin-1, occludin, and zonula Occludens-1 (ZO-1)). Furthermore, DP71L treatment markedly reduces the levels of pro-inflammatory cytokines, alongside a concurrent increase in the anti-inflammatory cytokine IL-10. Collectively, these findings establish DP71L as a novel suppressor of colitis severity in mice and reveal its previously unrecognized potential as an immunosuppressant. This work not only expands the functional repertoire of ASFV-encoded proteins but also opens a new conceptual avenue for treating immune-mediated diseases by repurposing viral immunomodulatory machinery.

## 2. Results

### 2.1. DP71L Protein Inhibits Weight Loss in DSS-Induced Colitis Mice

**Figure 1:**
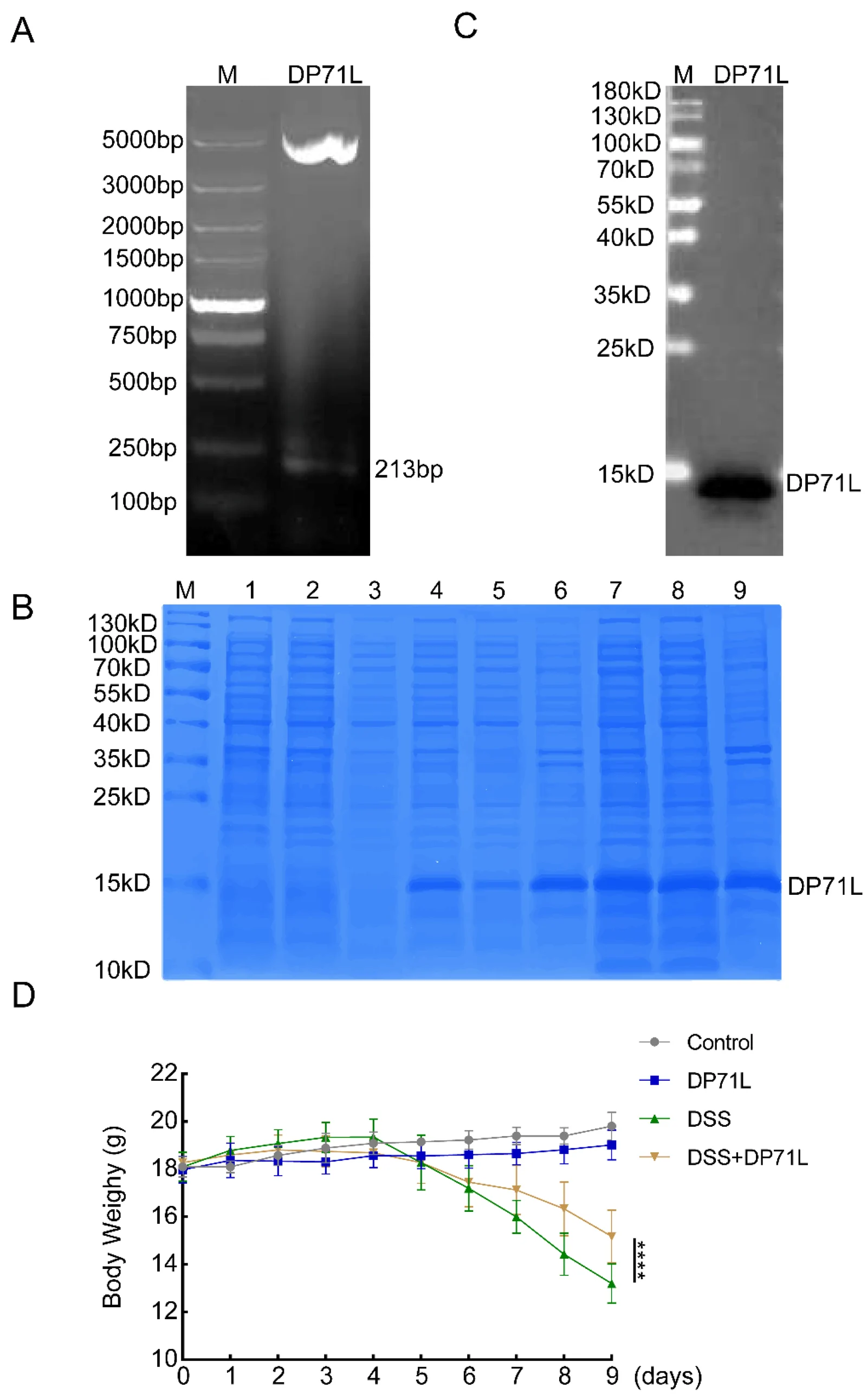
Purified DP71L protein alleviates weight loss in DSS-induced colitis mice. (**A**) M: DNA marker, pET28-DP71L plasmid confirmed by enzyme digestion (DP71L: 213 bp). (**B**) Purification of DP71L inclusion bodies post-renaturation. M: protein marker; 1: effluent solution; 2–4: miscellaneous protein eluted with 250 mM imidazole; 5–9: DP71L protein eluted with 500 mM imidazole. (**C**) Identification of purified DP71L protein via Western blotting with anti-His antibody. (**D**) Impact of DP71L on body weight in enteritis mice (*n* = 6 mice per group). Data are presented as mean ± SEM (standard error of the mean). Statistical significance was determined by one-way ANOVA. **** *p* < 0.0001.

DSS-induced ulcerative colitis causes pathological symptoms in mice, such as diarrhea, weight loss, colon shortening, and rectal bleeding. To evaluate the effect of DP71L protein on DSS-induced IBD in mice, we first successfully constructed the pET28-DP71L plasmid (Figure 1A). Then, the DP71L-His protein was purified and identified (Figure 1B,C). Meanwhile, a mouse colitis model was established with a 2% DSS solution. Furthermore, the effects of DP71L intervention on weight changes in colitis mice were recorded and analyzed. As shown in Figure 1D, although there was slight fluctuation in the body weight of the control group and the DP71L treatment group, an overall stable upward trend was observed without significant differences. This suggests that the dosage of the DP71L protein injected did not cause harm to the mice and had no apparent toxic side effects. The body weight of the DSS-treated mice began to decline on day 5 post-intervention and decreased sharply thereafter, indicative of severe intestinal injury. The body weight of the mice in the DSS-plus-DP71L group also started to decrease on the 5th day, but the rate of weight loss was significantly slower compared to the DSS group. This suggests that DP71L can slow down the weight loss of colitis mice.

### 2.2. DP71L Protein Inhibits Organ Damage in Mice with Colitis

**Figure 2:**
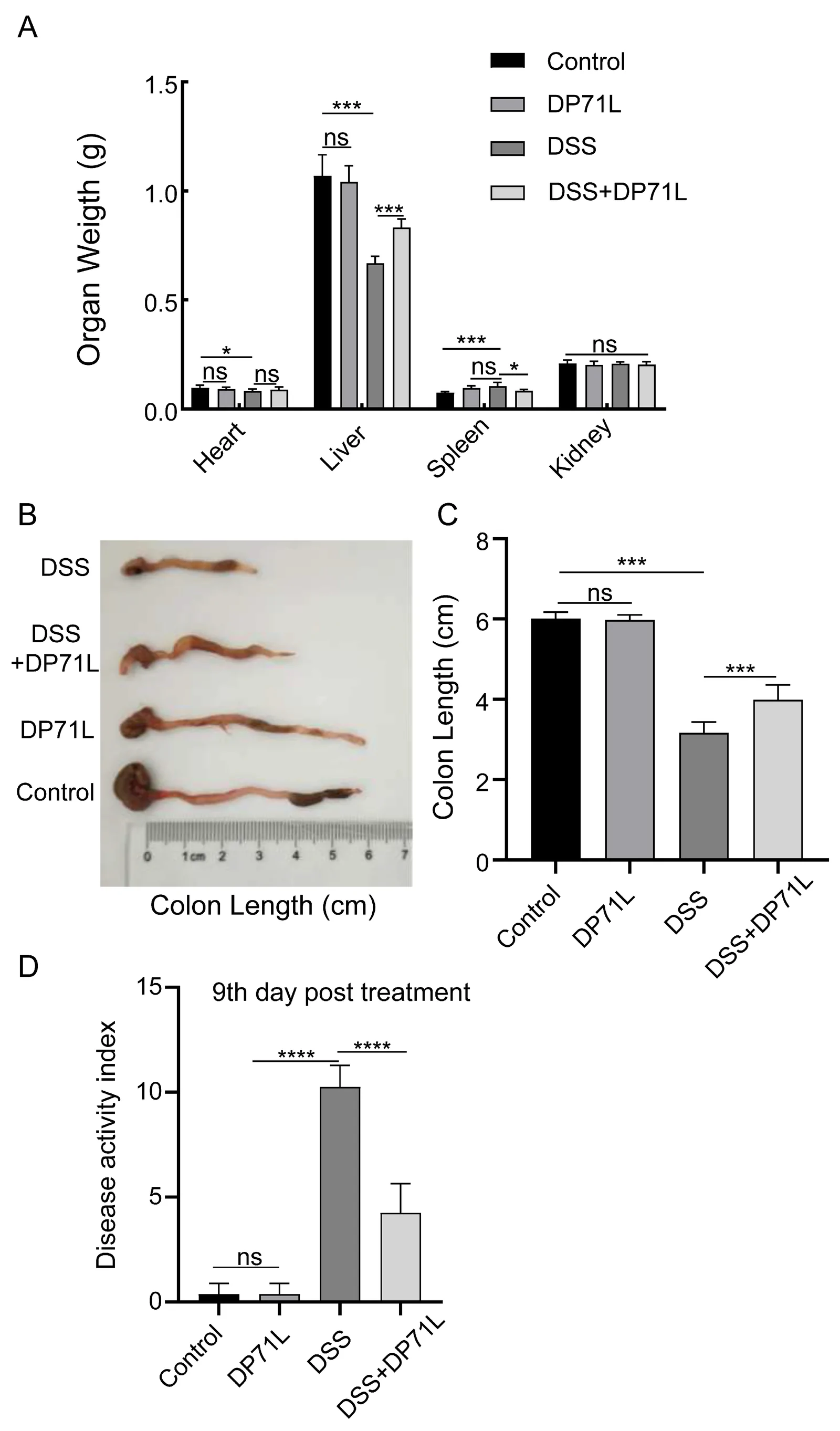
DP71L protein inhibits organ damage in colitis mice. (**A**) Influence of DP71L on organ weight in colitis mice. The weights of the heart, liver, spleen, and kidney were measured and compared. (**B**) Colon length measurement post-sacrifice and dissection of mice 10 days after modeling. (**C**) Statistical analysis of the differences in colon length among mice in different groups. (**D**) DAI for colitis in various treatment groups. Data are presented as mean ± SEM (standard error of the mean). Statistical significance was determined by *t*-test or one-way ANOVA. * *p* < 0.05; *** *p* < 0.001; **** *p* < 0.0001. ns (not significant). All experiments were repeated independently at least three times with similar results.

To evaluate the effect of DP71L on DSS-induced organ damage, hearts, livers, spleens, and kidneys were weighed. As shown in Figure 2A, compared with the control group, the liver weight of the DSS-treated mice significantly decreased (*p* < 0.001), while the spleen weight significantly increased (*p* < 0.001). DP71L intervention significantly attenuated the DSS-induced decrease in liver weight (*p* < 0.001) and the increase in spleen weight (*p* < 0.05).

Colon shortening is one of the typical symptoms of IBD and an important indicator to assess the severity of colitis. The results, shown in Figure 2B,C, show that the colon length of the mice in the control and DP71L-treated groups was essentially similar, indicating that the DP71L protein did not affect the colon length of the mice. Compared with the control group, the DSS-treated group exhibited significant colon shortening (*p* < 0.001), indicating that DSS caused intestine damage in mice. However, compared with the DSS group, the colon shortening in the DSS + DP71L group was alleviated (*p* < 0.001). The Disease Activity Index (DAI) score is extensively used to assess the severity and development of colitis. Compared with DSS-treated mice, the DSS + DP71L treatment significantly reduced the DAI score (Figure 2D). These results indicated that DP71L confers protective effects against DSS-induced colitis in mice.

### 2.3. DP71L Protein Inhibits Colonic Tissue Pathological Damage in Colitis Mice

**Figure 3:**
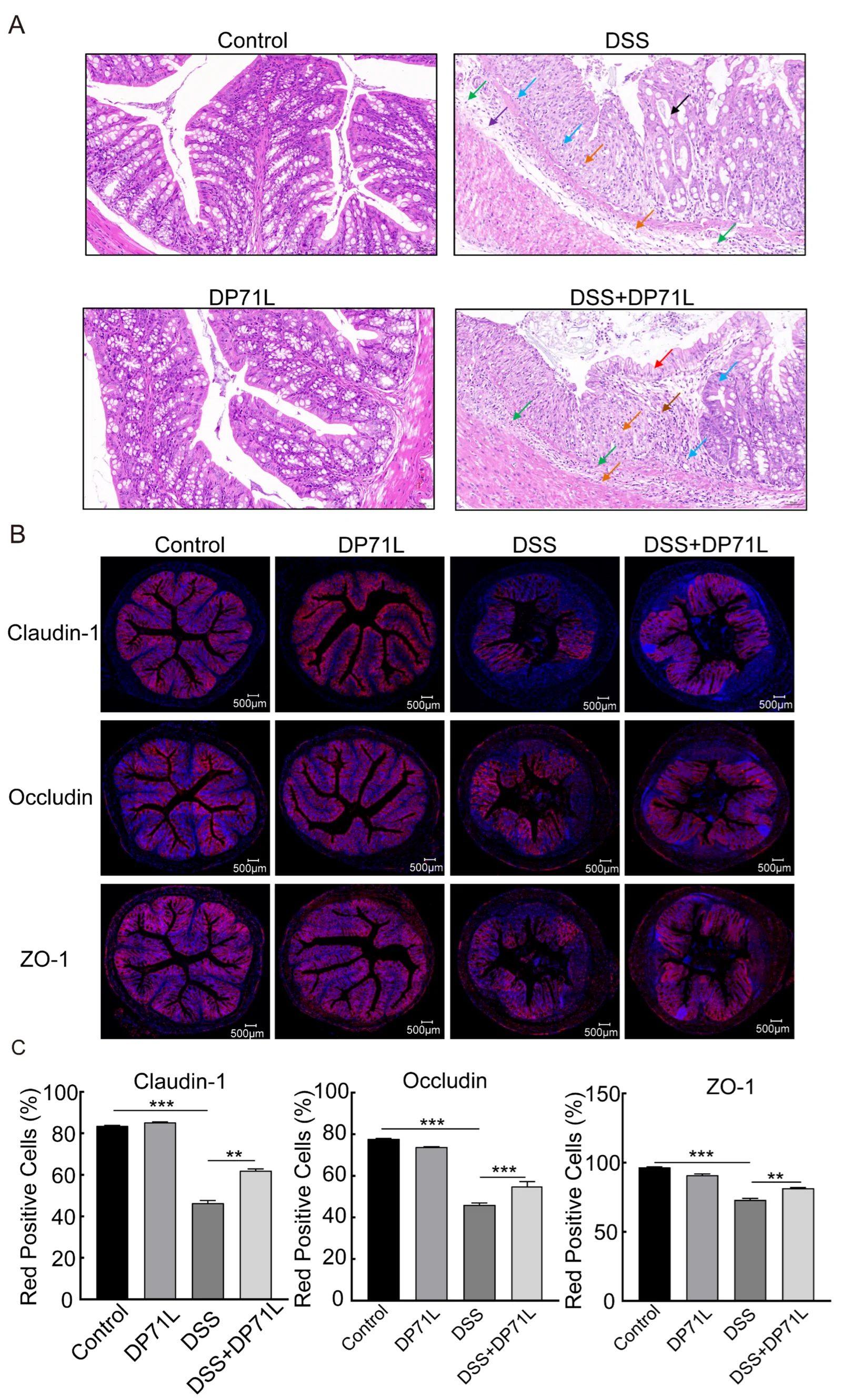
DP71L protein reduces colonic tissue pathological damage in colitis mice. (**A**) Effect of DP71L on histopathological morphology of colonic tissues in mice with colitis. Connective tissue proliferation (orange arrow); infiltration of lymphocytes, granulocytes and macrophages (blue/green arrows); intestinal gland dilation (black arrow); submucosal edema (purple arrow); mild connective tissue proliferation in the submucosa (orange arrow); hydropic degeneration of mucosal epithelial cells (red arrow); intestinal gland enlargement (brown arrow). (**B**,**C**) Effect of DP71L on tight-junction proteins in mice with colitis. (**B**) Fluorescence images of tight-junction proteins in mouse colons (primary antibody 1:200). (**C**) Quantitative analysis of claudin-1, occludin, and ZO-1 proteins’ fluorescence intensity under different treatments. Data are presented as mean ± SEM (standard error of the mean). Statistical significance was determined by *t*-test or one-way ANOVA. ** *p* < 0.01; *** *p* < 0.001. All experiments were repeated independently at least three times with similar results.

IBD can lead to intestinal tissue damage. Histological sections of mouse colon tissue were examined, and as shown in Figure 3A, no inflammatory cell infiltration was observed in the intestinal tissue of the control group and the DP71L-only group; the glands were regularly arranged, and the structure of epithelial cells and crypt was intact. In the DSS-treated group, pathological changes including submucosal edema, a reduced number of intestinal glands, connective tissue hyperplasia, and scattered infiltration of lymphocytes, granulocytes and macrophages were observed. Compared with the DSS group, the DSS + DP71L group had alleviated pathological changes, indicating that the DP71L protein had an inhibitory effect on colonic inflammation in the IBD mice.

As the most critical and fundamental mode of intercellular connection in the intestine, tight junctions play a key role in maintaining the epithelial physical barrier function and mucosal permeability [12]. Their proteins, known as tight-junction proteins, are crucial for regulating intestinal wall permeability and forming the protective mucosa layer [13]. Among them, claudin-1, occludin and ZO-1 are key tight-junction proteins that contribute to maintaining cell polarity and the epithelium mucosal physical barrier [14]. The fluorescence intensities of claudin-1, occludin, and ZO-1 were quantified and statistically analyzed. Compared with the normal group, the fluorescence intensity of claudin-1 (*p* < 0.001), occludin (*p* < 0.001), and ZO-1 (*p* < 0.001) in the DSS group was significantly decreased. However, DP71L treatment inhibited the loss of claudin-1 (*p* < 0.01), occludin (*p* < 0.001), and ZO-1 (*p* < 0.01) (Figure 3B,C). These results further confirm that DSS attenuates the expression of tight-junction proteins in the intestinal physical barrier, thereby increasing intestinal permeability, whereas DP71L intervention prevents the loss of tight-junction proteins and protects against colonic tissue damage in colitis mice.

### 2.4. DP71L Protein Inhibits Oxidative Stress in Colitis Mice

**Figure 4:**
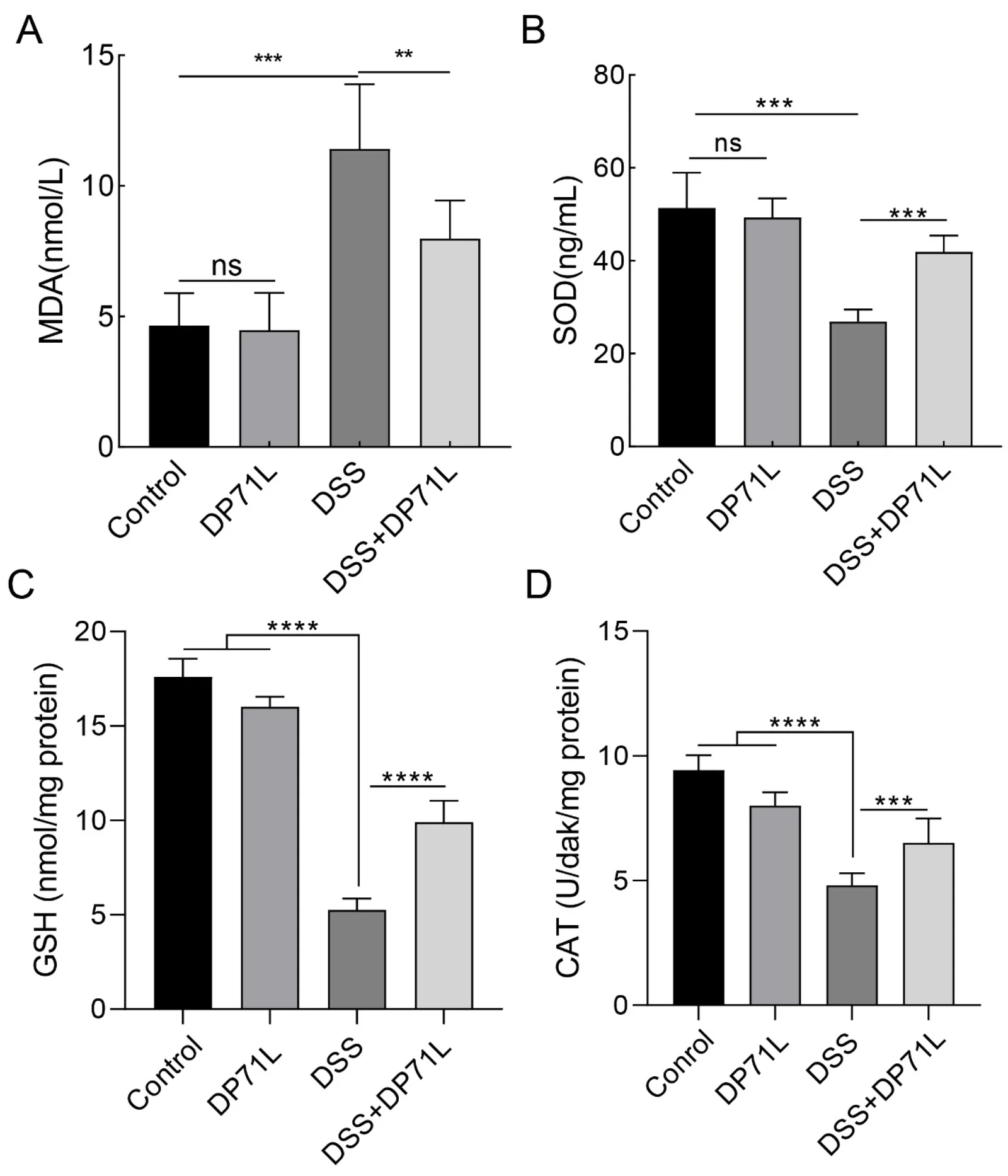
DP71L Protein inhibits oxidative stress in colitis mice. Protein levels of (**A**) MDA, (**B**) SOD, (**C**) GSH and (**D**) CAT in colon tissue homogenates were measured by ELISA. Colon tissues (0.1 g) were homogenized in PBS (1:9) containing protease inhibitors (1:100), and supernatants were collected for ELISA analysis. Data are presented as mean ± SEM (standard error of the mean). Statistical significance was determined by t-test or one-way ANOVA. ** *p* < 0.01; *** *p* < 0.001; **** *p* < 0.0001. ns (not significant). All experiments were repeated independently at least three times with similar results.

The accumulation of reactive oxygen species (ROS) and the weakened antioxidant defense can induce oxidative stress, damaging proteins and lipids, releasing cytokines, and triggering inflammation, which further exacerbate cell damage [14]. During oxidative stress, ROS increase free radicals in endothelial cells and reduce the activity of scavenging enzymes such as superoxide dismutase (SOD). Consequently, oxygen free radicals accumulate within cells, causing oxidation of unsaturated fatty acids in the bio-membrane and the production of malondialdehyde (MDA) [15]. We also measured catalase (CAT) and glutathione (GSH) activities to evaluate oxidative stress. As shown in Figure 4A,B, DSS-induced colitis mice exhibited significantly reduced SOD activity (*p* < 0.001) and increased MDA level (*p* < 0.001) compared to the control group. However, DP71L treatment significantly increased SOD activity (*p* < 0.001) and decreased MDA levels (*p* < 0.01). Moreover, CAT and GSH activities were significantly higher in the DP71L + DSS group than in the DSS group (Figure 4C,D). These data indicate that DP71L reduces oxidative stress in colitis mice.

### 2.5. DP71L Protein Inhibits Pro-Inflammatory Cytokine Production in Colitis Mice

**Figure 5:**
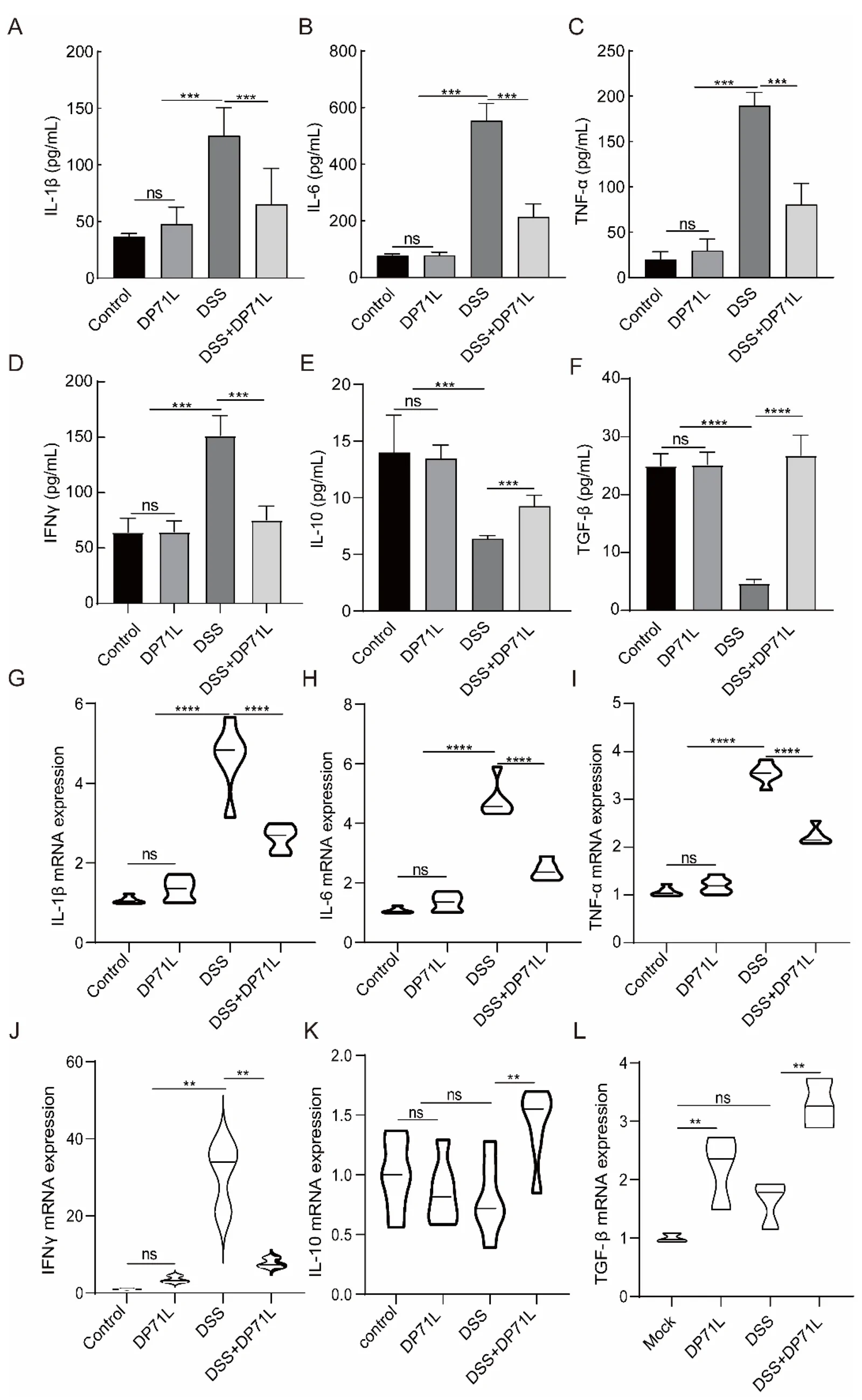
DP71L protein inhibits the production of pro-inflammatory cytokines in colitis mice. (**A**–**F**) Protein levels of IL-1β, IL-6, TNF-α, IFN-γ, IL-10, and TGF-β in colon tissue homogenates were measured by ELISA. Colon tissues (0.1 g) were homogenized in PBS (1:9) containing protease inhibitors (1:100), and supernatants were collected after centrifugation at 5000 r/min for 15 min at 4 °C. The supernatant was utilized for TNF-α, IL-1β, IL-6, and IL-10 ELISAs following the kit’s instructions. (**G**–**L**) Relative mRNA expression of IL-1β, IL-6, TNF-α, IFN-γ, IL-10, and TGF-β was analyzed by qPCR and normalized to the internal control. Data are presented as mean ± SEM (standard error of the mean). Statistical significance was determined by Student’s *t*-test or one-way ANOVA. ** *p* < 0.01; *** *p* < 0.001; **** *p* < 0.0001. ns (not significant). All experiments were repeated independently at least three times with similar results.

Cytokines can have pro-inflammatory effects, such as TNF-α, IL-6, IFN-γ, and IL-1β, or anti-inflammatory effects, such as IL-10 and TGF-β. To evaluate the regulatory effect of DP71L on inflammatory cytokines, we measured the colonic levels of TGF-β, IL-10 and IFN-γ, IL-1β, IL-6 and TNF-α. As shown in Figure 5A–F, compared with the control group, the DSS group exhibited significantly increased secretion of the pro-inflammatory cytokines IL-1β (*p* < 0.001), IL-6 (*p* < 0.001), IFN-γ (*p* < 0.001), and TNF-α (*p* < 0.001), as well as significantly decreased IL-10 and TGF-β levels (*p* < 0.001). However, DP71L intervention significantly reduced the levels of IL-1β (*p* < 0.001), IL-6 (*p* < 0.001), IFN-γ (*p* < 0.001), and TNF-α (*p* < 0.001), whereas it increased the levels of IL-10 (*p* < 0.001) and TGF-β (*p* < 0.0001) in the colonic tissue of colitis mice. Consistently, the mRNA expression of IL-1β, IL-6, IFN-γ, and TNF-α was decreased after DP71L treatment compared with the DSS group, but IL-10 and TGF-β increased (Figure 5G–L). These data indicated that DP71L suppressed pro-inflammatory cytokine production in mice with colitis.

### 2.6. DP71L Inhibits DSS-Triggered Innate Immunity and Apoptosis

In patients with ulcerative colitis (UC), the phosphorylation levels of JAK2 and STAT3 are markedly elevated in inflamed colonic mucosa [16], and enhanced JAK2/STAT3 phosphorylation exacerbates colitis [17]. In DSS-induced colitis, the NF-κB signaling pathway is one of the most critical drivers of inflammation, as DSS treatment increases IκBα phosphorylation and promotes nuclear translocation of NF-κB p65 in colonic tissue [18]. DSS induces apoptosis through multiple pathways [19,20,21,22], whereas DP71L is recognized as a key anti-apoptotic protein encoded by African swine fever virus (ASFV) [23].

**Figure 6:**
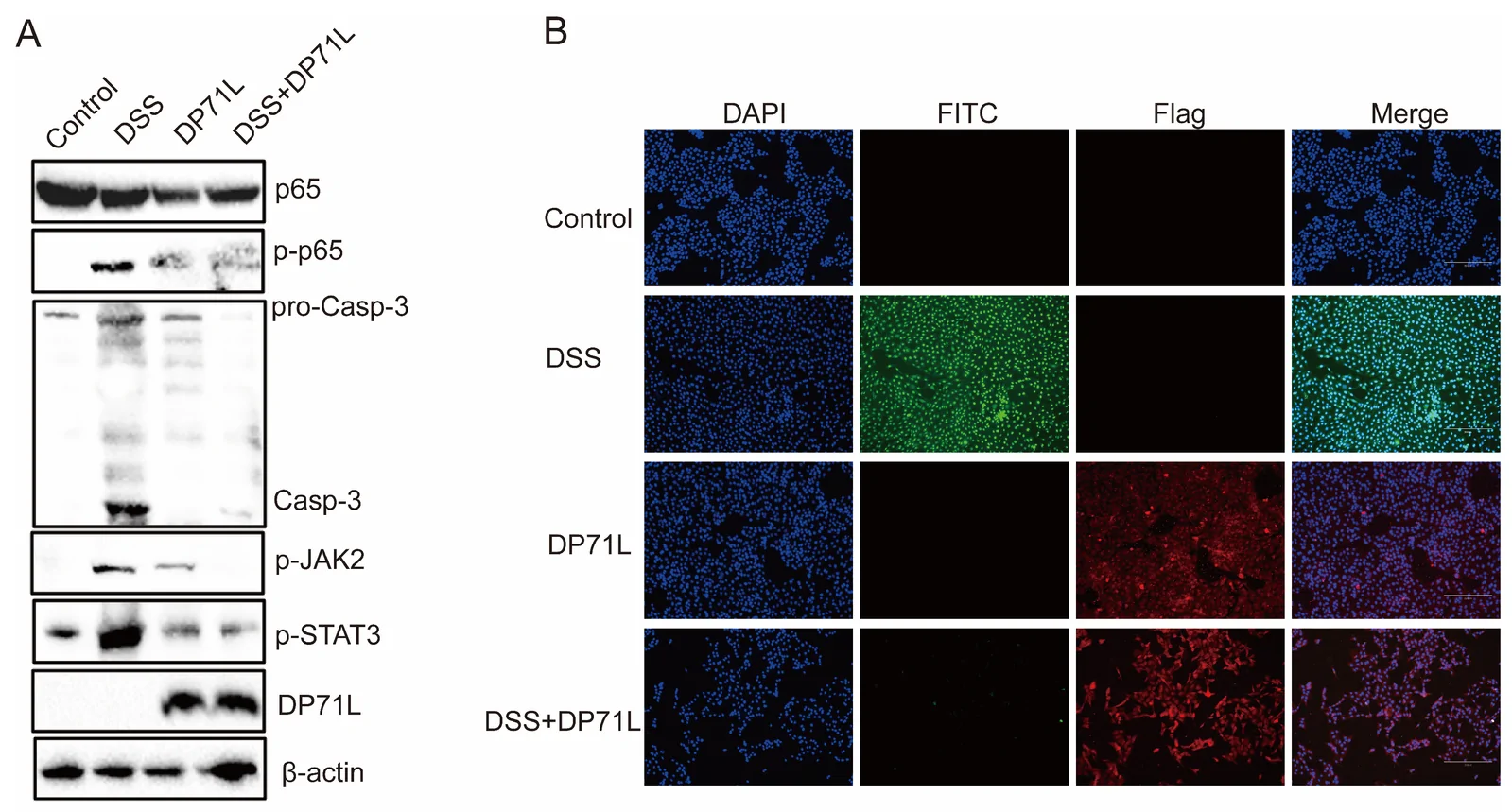
DP71L inhibits DSS-triggered innate immunity and apoptosis. (**A**) MODE-K cells were treated with DSS, DP71L, or DSS + DP71L. Phosphorylation levels of p65, JAK2 and STAT3 were examined, and cleavage of caspase-3 was assessed by Western blotting. (**B**) MODE-K cells were treated with DSS, DP71L, or DSS plus DP71L. Apoptosis was detected by TUNEL assay and visualized using fluorescence microscopy, broken DNA (green), DP71L-Flag (red).

We therefore examined the effects of DP71L on DSS-induced NF-κB, JAK2, STAT3 and apoptotic signaling. Our data showed that DSS upregulated the phosphorylation of NF-κB, JAK2, and STAT3 and increased the expression of active caspase-3 (Casp-3), an apoptosis marker, while fluorescence assays revealed extensive intestinal cell death. Conversely, DP71L treatment reduced the phosphorylation of these inflammatory signaling molecules (NF-κB, JAK2 and STAT3) and inhibited DSS-induced apoptosis (Figure 6A,B). Collectively, these findings indicate that DP71L suppresses DSS-triggered inflammatory responses and apoptosis.

## 3. Discussion

UC is a type of chronic inflammatory bowel disease (IBD) that has become a global disease. As the prevalence of UC increases, traditional drugs often fail to achieve desired therapeutic effects due to severe side effects [24]. Therefore, there is an urgent need to develop an effective IBD treatment with minimal or no side effects.

This study establishes that ASFV-derived DP71L protein ameliorates DSS-induced murine colitis through a suppression of NF-κB and JAK-STAT signaling cascades alongside inhibition of Caspase-3-mediated epithelial apoptosis. Mechanistically, DP71L significantly reduced phosphorylation of NF-κB p65, JAK2, and STAT3 in colonic cells, thereby attenuating transcription of key pro-inflammatory cytokines (IL-6, IL-1β, TNF-α, and IFN-γ) while preserving anti-inflammatory IL-10 and TGF-β levels. Concurrently, Caspase-3 inhibition mitigated intestinal epithelial cell apoptosis—a critical driver of barrier disruption in IBD [25,26]. This dual-pathway modulation directly correlates with restored expression of tight-junction proteins (claudin-1, occludin, ZO-1), reinforcing mucosal integrity. Notably, ZO-1 deficiency alone impairs mucosal healing independent of tight-junction assembly, underscoring its non-redundant role in epithelial repair [27,28,29]; DP71L’s capacity to sustain ZO-1 expression thus addresses a fundamental deficit in UC pathophysiology.

Compared to current IBD therapeutics, DP71L exhibits a distinctive multi-target profile. While anti-TNF-α biologics (e.g., infliximab) and selective JAK inhibitors (e.g., upadacitinib) target single inflammatory axes [30], DP71L concurrently modulates NF-κB, JAK-STAT, and apoptotic pathways—mirroring the efficacy of multi-mechanistic natural compounds like Astragalin (which degrades FPR1 to suppress NF-κB) [31,32] or CAP polysaccharides (activating MAPK/NF-κB for barrier restoration) [33].

Translational development requires addressing delivery and safety challenges. Shifting from intraperitoneal injection to oral administration presents theoretical advantages for clinical feasibility but encounters substantial physiological barriers. Orally delivered protein therapeutics must overcome gastric acidity, proteolytic degradation, and limited intestinal epithelial permeability. Inspired by successful colon-targeted systems (e.g., OCMC/SA nanohydrogels protecting insulin, or stereoisomer-engineered dipeptides exploiting leaky tight junctions in an inflamed colon) [34,35,36], future work will: (1) identify minimal bioactive DP71L peptide motifs via structural mapping; (2) conjugate these motifs to pH/enzyme-responsive nanocarriers (e.g., mesoporous silica 13 or lipid-based systems 14) for site-specific release [37]; and (3) incorporate permeation enhancers to leverage inflammation-induced paracellular transport [38].

Concurrently, rigorous biosafety assessment is essential. Although DP71L is a purified recombinant protein lacking viral infectivity, and prior studies utilized ASFV proteins (e.g., DP238L, DP71L) as safe vaccine antigens [39] (Chinese patent, patent number: CN116019905A), rigorous assessment of off-target immunogenicity remains essential. We will evaluate antigenicity, allergenicity, and dose-dependent toxicity per framework for biologic safety profiling. Notably, DP71L’s established role as a potent integrated stress response (ISR) inhibitor in neurological models [40] warrants investigation into tissue-specific effects, though its barrier-protective actions in colitis align with ISR modulation benefits observed in other inflammatory contexts.

This study has limitations. First, efficacy was assessed at a single dose via intraperitoneal injection; future studies must establish dose–response relationships and compare oral/rectal delivery routes. Second, control groups lacked vehicle-only or affinity-tag (e.g., His-tag) controls to exclude nonspecific effects of the expression system. Third, causal validation of NF-κB/JAK-STAT inhibition requires genetic models (e.g., STAT conditional KO) or pathway-specific inhibitors (e.g., AG490 for JAK2 [41]. Finally, the long-term immunogenicity and impact on gut microbiota composition (critical for therapies like PSV or BB1 that exert effects via microbial modulation [42,43]) warrant investigation.

In conclusion, DP71L represents a novel virally derived immunomodulator that concurrently targets inflammatory signaling and epithelial apoptosis—core pathological pillars of UC. Its multi-mechanistic action offers a rational strategy to overcome the limitations of single-target biologics. Future efforts will focus on peptide minimization, colon-targeted delivery engineering, and comprehensive biosafety validation. While repurposing pathogen-derived proteins demands cautious risk–benefit evaluation, disciplined molecular refinement may unlock a new class of precision therapeutics for IBD, exemplifying how viral immune evasion mechanisms can be ethically harnessed for host-directed anti-inflammatory therapy.

## 4. Materials and Methods

### 4.1. Animals and Reagents

Specific pathogen-free (SPF) female C57BL/6 mice, aged 6 weeks, were purchased from the Lanzhou Veterinary Research Institute, Chinese Academy of Agricultural Sciences. In this study, animal experiments were performed in accordance with the Guide for the Care and Use of Laboratory Animals of the Ministry of Science and Technology of China. All animal experimental procedures were approved by the Northwest Minzu University Animal Ethics Committee (approval number: xbmu-sm2024031). Mouse IL-10, IL-1β, IL-6, IFN-γ, TGF-β, and TNF-α enzyme-linked immunosorbent assay (ELISA) kits were purchased from Shenzhen Xin bo sheng Biotechnology Co., Ltd. (Shenzhen, China). Mouse SOD and MDA ELISA kits were purchased from Xiamen LUNCHANGSHUO Biotechnology Co., Ltd. (Xiamen, China). DSS was obtained from Shanghai Biyun Biotechnology Co., Ltd. (Shanghai, China). The One-Step TUNEL Apoptosis Assay Kit (green fluorescence, C1086) was aquired from Beyotime (Shanghai, China). Monoclonal anti-FLAG M2 mouse antibody and the Anti-beta-Actin antibody were purchased from Sigma-Aldrich ((St. Louis, MO, USA). Anti-mouse IgG (H + L) F(ab′)_2_ Fragment (Alexa Fluor 594 Conjugate) and NF-kappaB p65 (D14E12) XP Rabbit mAb and p-NF-kappaB p65 (S536) (93H1) Rabbit mAb were aquired from Cell Signaling Technology (Danvers, MA, USA). Fetal Bovine Serum (Aoqing Biotechnology Co., Ltd., Beijing, China)was used. DAPI Nuclear Staining Solution (Beyotime, Shanghai, China) was used. The DP71L antibody and MODE-K cells were stored in our lab. Phospho-STAT2-Y690 Rabbit pAb, Phospho-STAT1-Y701 Rabbit mAb, Phospho-STAT3-S727 Rabbit pAb, and Phospho-JAK2-Y1007/1008 Rabbit pAb were aquired from Abclonal (Wuhan, China). Horseradish peroxidase (HRP)-conjugated goat anti-rabbit IgG (H + L) and goat anti-mouse IgG (H + L) antibodies were from Zhongshan Golden Bridge Biotechnology (Beijing, China). WesternBright ECL Chemiluminescent Substrate was from Advansta (Mountain View, CA, USA). The 0.22 µm PVDF membrane was from Merck-Millipore (Darmstadt, Germany).

### 4.2. Plamid Construction and Expression

Using the African swine fever virus genome (ASFV/HLJ/18) as a template, the DP71L gene was synthesized and cloned into the prokaryotic expression vector pET28a (+), followed by sequence confirmation. The recombinant DP71L protein was expressed in *E. coli* BL21 cells and purified. The purified protein was dissolved in PBS (phosphate-buffered saline) for subsequent immunoassays.

### 4.3. Establishment of Colitis Model

Thirty-two mice were randomly divided into four groups: control group, DP71L group, DSS group, and DSS + DP71L group. Each mouse was individually marked. The DSS group and DSS + DP71L group were given drinking water containing 2% DSS, while the control group and DP71L group were given normal drinking water. After 7 days, the DSS group and DSS + DP71L group were switched to normal drinking water. On days 1, 3, 5, 7, and 9, the DP71L group and DSS + DP71L group were intraperitoneally injected with 10 μg (100 μg/mL, 100 μL) recombinant DP71L protein (once every other day). The mice were euthanized ten days later. Throughout the experiment, the body weight and general condition of the mice were recorded daily.

### 4.4. Collection of Tissue Samples

The colon tissues from the mice were collected, and the colon length was measured. The tissues were then fixed, and sections were prepared. The remaining colon tissues were stored at −80 °C. The heart, liver, kidneys, and spleen of the mice were weighed.

### 4.5. Histopathological Analysis of Colon Tissues

The sections of mouse colon were stained with hematoxylin and eosin to observe histological changes. The colon tissues were fixed in 4% paraformaldehyde for 48 h, dehydrated in ethanol, cleared in xylene, and embedded in paraffin. The paraffin-embedded tissues were cut into 5 μm thick sections, deparaffinized, and dried. The sections were then stained with hematoxylin and eosin and examined under a microscope.

### 4.6. Immunofluorescence Staining of Colon Tissues

The deparaffinized sections were rehydrated and subjected to antigen retrieval. After incubation with BSA (bovine serum albumin), the sections were incubated with primary antibodies against claudin (1:200), ZO-1 (1:200), and occludin (1:200) overnight at 4 °C, followed by washing with PBS. The sections were then incubated with a secondary antibody (goat anti-rabbit IgG) at room temperature in the dark for 1 h. After drying, the sections were stained with DAPI (4′,6-diamidino-2-phenylindole), incubated in the dark for 10 min, and treated with a self-quenching reagent for 5 min. Finally, the sections were mounted and observed under a fluorescence microscope for analysis and photography.

### 4.7. Antioxidant Index and Cytokine Detection

After removing the residual feces, the tissue was cut into pieces. The corresponding PBS was added according to a ratio of tissue to PBS of 1:9, and protease inhibitors were added to PBS at a ratio of 1:100. The ground tissue homogenate was fully crushed again using an ultrasonic crusher, and finally the homogenate was centrifuged at 5000 r/min for 15 min at 4 °C. The supernatant was removed and tested according to the ELISA kit instructions.

### 4.8. RNA Extraction and Real-Time PCR

Take 0.1 g of mouse colon tissue; rinse it thoroughly with pre-cooled PBS to remove any remaining feces. Cut the tissue into small pieces and add PBS at a ratio of 1:9, along with protease inhibitors at a ratio of 1:100. Homogenize the tissue thoroughly at low temperature using a tissue crusher, followed by additional disruption using an ultrasonic crusher. Add TRIzol reagent (Invitrogen, Thermo Fisher Scientific, Carlsbad, CA, USA) to extract total RNA following the manufacturer’s instructions. Synthesize cDNAs using reverse transcriptase (Promega, Madison, WI, USA) and random hexamer primers. Perform quantitative real-time PCR analysis using an Mx3005P qPCR system (Agilent Technologies, Santa Clara, CA, USA) with SYBR premix Ex Taq reagents (TaKaRa Biomedical Technology (Beijing) Co., Ltd., Beijing, China)). Calculate the relative fold changes of mRNA using the comparative cycle threshold (2^−ΔΔCT^) method. Design and refer to Table 1 for the gene-specific primers for *TNF-α*, *IL-1β*, *IL-6*, *IL-10*, and *GAPDH*.

### 4.9. One-Step TUNEL Apoptosis Assay

MODE-K cells were seeded in confocal culture dishes and cultured until they reached approximately 50% confluence. Cells were then treated with DP71L protein solution or DSS (20 mg/mL) for 48 h. After treatment, cells were washed and fixed with 4% paraformaldehyde for 15 min at room temperature. Following removal of the fixative, cells were permeabilized with 0.1% Triton X-100 for 20 min at room temperature. After discarding the permeabilization solution, cells were blocked with 10% FBS for 1 h at 37 °C. Then, the primary antibody (anti-FLAG) was added and incubated overnight at 4 °C. The primary antibody was removed, and cells were washed four times with PBST, for 5 min each wash. Subsequently, the fluorescently labeled secondary antibody was added and incubated for 45 min at room temperature in the dark. After removal of the secondary antibody, cells were washed four times with PBST (5 min each) under dark conditions. The TUNEL reaction mixture (TdT enzyme 50 µL: fluorescent labeling solution 450 µL) was prepared and thoroughly mixed. Then, 100 µL of this mixture was applied to each sample and incubated for 60 min at 37 °C in the dark. The TUNEL mixture was then removed, and cells were washed four times with PBST (5 min each) in the dark. Afterwards, DAPI was added and incubated for 10 min at room temperature in the dark. The DAPI solution was discarded, and cells were washed three times with PBST (3 min each) under dark conditions. Finally, a small volume of PBST was added to cover the cells, and images were acquired using a fluorescence microscope.

### 4.10. Western Blotting

MODE-K cells were cultured in RPMI 1640 complete medium at 37 °C in a 5% CO_2_ incubator. When cells reached approximately 70% confluence, they were treated with DP71L or DSS (20 mg/mL) for 48 h. Cells were then harvested, washed, and lysed. The lysates were centrifuged at 12,000 r/min, and the supernatants were collected. A 4× protein loading buffer was added, and samples were boiled for 10 min. Equal amounts of protein were loaded and separated by SDS-PAGE at 80 V for 30 min, followed by 120 V for 45 min. After electrophoresis, proteins were transferred onto PVDF membranes (pre-activated with methanol for 15 s and equilibrated in transfer buffer for 5 min) using a wet-transfer system at 100 V for 90 min. Membranes were blocked with 5% (*w*/*v*) non-fat milk in TBST for 4 h at 4 °C with gentle shaking. After blocking, membranes were incubated with primary antibodies (diluted in primary antibody diluent) overnight at 4 °C with slow shaking, then washed five times with TBST (5 min each). Membranes were then incubated with HRP-conjugated secondary antibodies (diluted in secondary antibody diluent) for 45 min at room temperature in the dark with gentle agitation, followed by five washes with TBST (5 min each). Finally, ECL chemiluminescent substrate was applied and incubated for 1 min in the dark, and signals were detected and analyzed using a high-resolution imaging system.

### 4.11. Statistical Analysis

All experiments were repeated at least three times independently. Statistical analysis was performed using GraphPad Prism 8.3 (GraphPad Software, San Diego, CA, USA) and unpaired two-tailed Student’s t-test or one-way ANOVA. *p* < 0.05 was considered statistically significant. * *p* < 0.05; ** *p* < 0.01; *** *p* < 0.001; *** *p* < 0.0001. ns, not significant.

## Figures and Tables

**Table 1:** The primers for cytokine qPCR detection.

Gene	Forward Primer (5′-3′)	Reverse Primer (5′-3′)
*IL-1β*	GAAATGCCACCTTTTGACAGTG	TGGATGCTCTCATCAGGACAG
*IL-6*	CTGCAAGAGACTTCCATCCAG	AGTGGTATAGACAGGTCTGTTGG
*TNF-α*	CAGGCGGTGCCTATGTCTC	CGATCACCCCGAAGTTCAGTAG
*IFN* *γ*	TGGCATAGATGTGGAAGAAAAGAG	TGCAGGATTTTCATGTCACCAT
*IL-10*	ATTTGAATTCCCTGGGTGAGAAG	CACAGGGGAGAAATCGATGACA
*TGF-β*	GTGTGGAGCAACATGTGGAACTCTA	TTGGTTCAGCCACTGCCGTA
*GAPDH*	AGAACATCATCCCTGCATCC	CACATTGGGGGTAGGAACAC

## Data Availability

The original contributions presented in the study are included in the article. Further inquiries can be directed to the corresponding authors.
